# Reactive Oxygen Species Are Key Mediators of Demyelination in Canine Distemper Leukoencephalitis but not in Theiler’s Murine Encephalomyelitis

**DOI:** 10.3390/ijms20133217

**Published:** 2019-06-30

**Authors:** Friederike Attig, Ingo Spitzbarth, Arno Kalkuhl, Ulrich Deschl, Christina Puff, Wolfgang Baumgärtner, Reiner Ulrich

**Affiliations:** 1 Department of Pathology, University of Veterinary Medicine Hannover, D-30559 Hannover, Germany; 2 Center for Systems Neuroscience, D-30559 Hannover, Germany; 3 Department of Non-clinical Drug Safety, Boehringer Ingelheim Pharma GmbH & Co.KG, D-88397 Biberach, Germany; 4 Institute for Veterinary Pathology, Faculty of Veterinary Medicine, University of Leipzig, D-04103 Leipzig, Germany

**Keywords:** canine distemper leukoencephalitis, Theiler’s murine encephalomyelitis, reactive oxygen species, malondialdehyde, E06, 8-hydroxyguanosine, superoxide dismutase, catalase

## Abstract

(1) Background: Canine distemper virus (CDV)-induced demyelinating leukoencephalitis (CDV-DL) in dogs and Theiler’s murine encephalomyelitis (TME) virus (TMEV)-induced demyelinating leukomyelitis (TMEV-DL) are virus-induced demyelinating conditions mimicking Multiple Sclerosis (MS). Reactive oxygen species (ROS) can induce the degradation of lipids and nucleic acids to characteristic metabolites such as oxidized lipids, malondialdehyde, and 8-hydroxyguanosine. The hypothesis of this study is that ROS are key effector molecules in the pathogenesis of myelin membrane breakdown in CDV-DL and TMEV-DL. (2) Methods: ROS metabolites and antioxidative enzymes were assessed using immunofluorescence in cerebellar lesions of naturally CDV-infected dogs and spinal cord tissue of TMEV-infected mice. The transcription of selected genes involved in ROS generation and detoxification was analyzed using gene-expression microarrays in CDV-DL and TMEV-DL. (3) Results: Immunofluorescence revealed increased amounts of oxidized lipids, malondialdehyde, and 8-hydroxyguanosine in CDV-DL while TMEV-infected mice did not reveal marked changes. In contrast, microarray-analysis showed an upregulated gene expression associated with ROS generation in both diseases. (4) Conclusion: In summary, the present study demonstrates a similar upregulation of gene-expression of ROS generation in CDV-DL and TMEV-DL. However, immunofluorescence revealed increased accumulation of ROS metabolites exclusively in CDV-DL. These results suggest differences in the pathogenesis of demyelination in these two animal models.

## 1. Introduction

Reactive oxygen species (ROS) are chemically reactive molecules, which can cause severe damage to a vast variety of either intra- or extracellularly located macromolecules [1]. ROS take part in signaling pathways [1], in inflammatory processes, and as by-products of cellular metabolism [2]. A high concentration of ROS may arise because of intensive stimulation of nicotinamide adenine dinucleotide phosphate (NADPH-) oxidase, in mitochondrial electron chain transport, through xanthine oxidase or various other enzymatic or non-enzymatic pathways (reviewed by [3]). Overwhelming ROS production causes oxidative stress, if the cells’ defense system cannot stave off [4]. For instance, ROS produced by activated microglia and macrophages, can directly inhibit the respiratory chain or induce mitochondrial dysfunction leading to lack of adenosine triphosphate (ATP) [1]. Moreover, ROS-induced cell death may result from oxidation of important cellular components such as DNA, RNA, proteins, and lipids [5]. Though it is nearly impossible to identify the individual reactive molecule that causes a lesion observable by light microscopic evaluation of a hematoxylin and eosin stained section (reviewed by [3]), it is possible to detect the characteristic metabolites of ROS-induced oxidative reactions by immunohistochemistry. The molecules most susceptible to oxidative damage are lipids. Especially polyunsaturated fatty acids and arachidonic acid are highly vulnerable, and their degradation leads to the formation of characteristic metabolites such as malondialdehyde (MDA), 4-hydroxynonenal, and oxidized fatty acids [6]. Furthermore, different metabolites of oxidized lipids can be detected by various specific antibodies [7,8,9,10,11]. One of these antibodies is a clone that was developed in apo E-deficient mice. This marker detects oxidized phospholipids and the clone is called E06 [8]. Besides lipids, nucleic acids such as DNA and RNA are also highly susceptible to ROS-damage, and here, RNA seems to be more vulnerable than DNA. DNA or RNA can for instance be oxidized by hydroxyl radicals. The most commonly used markers for ROS-induced damage to DNA and RNA are 8-hydroxydeoxyguanosine (8-OHdG) and 8-hydroxyguanosine (8-OHG), respectively [6]. These products result from an oxidation of the carbon in the 8th position of the nucleoside guanosine [6]. Formation of 8-OHdG most commonly results from free radical-induced damage to DNA [12], and increases in both 8-OHdG/8-OHG generally point to an increase in free radical occurrence [12].

ROS can contribute to central nervous system (CNS) demyelination due to a direct effect of ROS on oligodendrocytes or myelin. Myelin is composed of lipids and proteins in which the lipid proportion represents at least 70% of dry weight [13,14,15], and ROS can damage both the protein and the lipid components of myelin [4].

Under normal circumstances, ROS generation is effectively counter-regulated by highly potent detoxification systems such as one of the three superoxide dismutases (SOD1-3) and catalase [16]. SODs trigger the catalyzation of superoxide (O_2_^• −^) into hydrogen peroxide (H_2_O_2_) [16]. Manganese SOD (MnSOD, also known as SOD2) is located in the inner mitochondrial matrix and is responsible for the reduction of superoxide production from the mitochondrial electron chain transport [16]. Catalase reduces H_2_O_2_ to water [6,17]. Catalase is located within the cytoplasm and is present in nearly every cell [18]. Neurons as well as glial cells within the CNS have been shown to express catalase [19].

Canine distemper virus (CDV) belongs to the genus *morbillivirus* and to the family *paramyxoviridae*. It is an enveloped negative-stranded RNA-virus and can cause a variety of clinical signs in dogs and other carnivores [18,20,21]. CNS involvement is common, and affected dogs suffer from ataxia and vestibular disease. Interestingly, CDV-induced demyelinating leukoencephalitis (CDV-DL) shares certain characteristics with other demyelinating diseases such as multiple sclerosis (MS) and its experimental animal models [21,22,23]. The viral antigen can be found in astrocytes, neurons, microglia, ependymal, leptomeningeal, and choroid cells, respectively. Virus replication begins within the lymphoid tissue with subsequent viremia [21], and possible viral spread within the brain via the cerebrospinal fluid (CSF) [24], and cell-to-cell spread using the astrocyte network [25]. The demyelination process during CDV-DL appears to occur in two subsequent phases. The first wave of demyelination is directly associated with the virus, where the virus is present within astrocytes, neurons, and possibly within oligodendrocytes [24,26,27]. In the second phase of the disease, demyelination is rather mediated by macrophages and/or antiviral antibodies [24,28]. In fact, oligodendrocytes are not the main target cell for infection with CDV [29]. Interestingly, in CDV-DL, demyelination takes place before the loss of oligodendrocytes [29,30]. This indicates that oligodendrocyte dystrophy as well as autoimmunity, mediated by immunoglobulin and complement, contribute to demyelination in CDV-DL. Substantiating this hypothesis, previous microarray data have revealed that genes related to the immune response mediated by immunoglobulin and the classical pathway of complement are up regulated in CDV-DL [31]. Furthermore, there was a downregulation of genes related to oligodendrocytes and myelin components [31].

Theiler’s murine encephalomyelitis virus (TMEV) belongs to the genus *cardiovirus* [32,33] within the *picornaviridae* family [34]. It is a positive, single-stranded RNA virus [32], which was first reported by Max Theiler [35]. The DA and BeAn strain of TMEV induce a biphasic disease in susceptible mouse strains, which affects predominantly the grey matter in the acute disease, followed by a chronic demyelinating disease especially in the spinal cord in advanced disease phases. While B6 mice are can clear the virus, SJL mice develop a chronic demyelinating disease in the spinal cord starting at 35-45 days post infection (dpi), mimicking primary progressive lesions of human MS [34,36].

The relevance of ROS for the pathogenesis of demyelination in CDV-DL and TMEV-induced demyelinating leukomyelitis (TMEV-DL) is currently unknown. Thus, the hypothesis of this study is that ROS are key effector molecules of myelin membrane breakdown in both CDV-DL and TMEV-DL. Therefore, our aims are (1) to evaluate the amount and cellular localization of ROS-induced metabolites, and (2) to analyze the transcriptional activity of important ROS-generating as well as detoxifying pathways in -CDV-DL and TMEV-DL, respectively [31,37].

## 2. Results

### 2.1. Lesion Profile in CDV-DL

**Figure 1:**
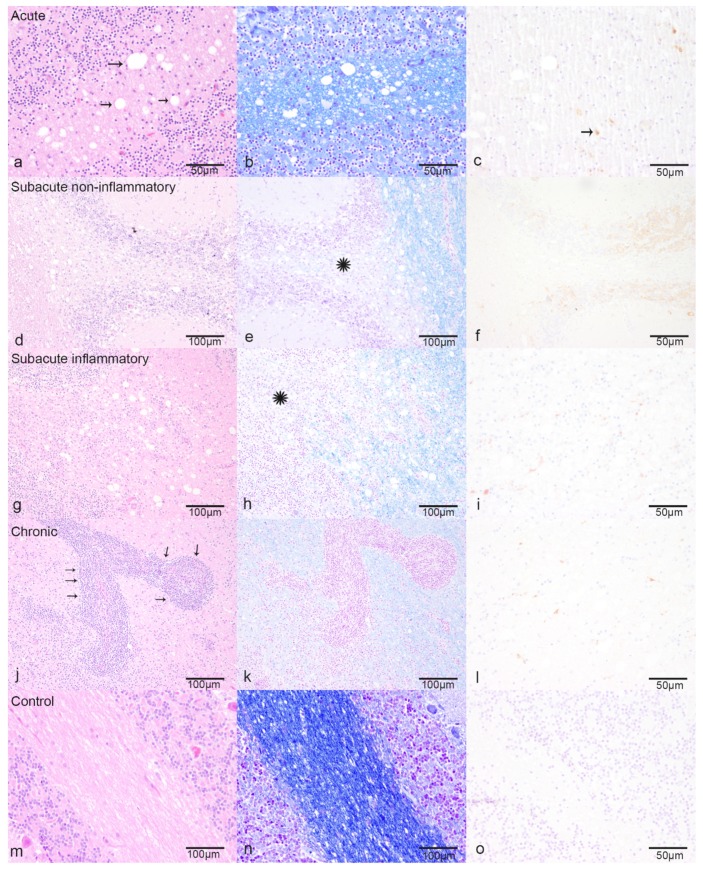
Characteristic pathohistological changes for the different subtypes of Canine distemper virus (CDV)-induced demyelinating leukoencephalitis (CDV-DL): (**a**–**c**) Acute CDV-DL lesions show multifocal dilated myelin sheaths (arrows), mild astrogliosis, and microgliosis, (**a**) Hematoxylin and eosin stain (HE), (**b**) Luxol fast blue (LFB), (**c**) immunohistochemistry for CDV-antigen positive cells (arrows), (**d**–**f**) Subacute non-inflammatory CDV-DL with beginning demyelination (LFB negative area, asterisk), astrogliosis and microgliosis, (**d**) HE, (**e**) LFB, (**f**) immunohistochemistry for CDV-antigen, (**g**–**i**) Subacute inflammatory CDV-DL with demyelination (LFB negative area, asterisk) and perivascular inflammatory infiltration up to three cell layers, (**g**) HE, (**h**) LFB, (**i**) immunohistochemistry for CDV-antigen, (**j**–**l**): Chronic CDV-leukoencephalitis with ongoing demyelination and severe perivascular inflammatory infiltration (arrows) more than three cell layers, (**j**) HE, (**k**) LFB, (**l**) immunohistochemistry for CDV-antigen, (**m**–**o**): Controls, (**m**) HE, (**n**) LFB, (**o**) immunohistochemistry for CDV-antigen.

**Figure 2:**
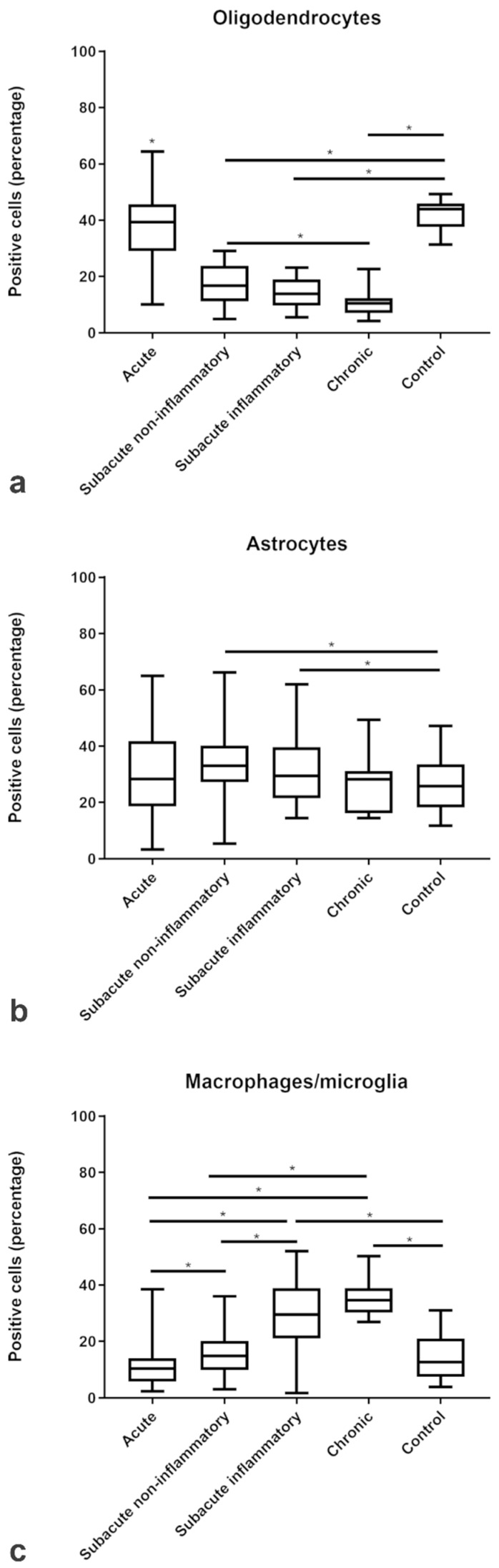
Relative changes in the proportions of (**a**) oligodendrocytes, (**b**) astrocytes, and (**c**) macrophages/microglia as compared to total intralesional cell counts in the different subgroups of CDV-DL and controls as assessed by immunohistochemistry for CNPase, GFAP, and Iba-1, respectively. Significant differences between the groups as revealed by the Kruskal–Wallis test with independent pairwise post-hoc Mann–Whitney U-test are marked with an asterisk (* *p* ≤ 0.05).

Each of the 16 CDV-infected dogs exhibited on average seven multifocal discrete lesions within the analyzed cerebellar FFPE specimen, as defined by histopathology and CDV-immunoreactivity (Figure 1, Supplementary Table S1). In contrast, the cerebellar specimen of all eight control dogs were immunonegative. The lesions were sub-grouped into acute CDV-DL (*n* = 49; Figure 1a–c), subacute non-inflammatory CDV-DL with demyelination (*n* = 26; Figure 1d–f), subacute inflammatory CDV-DL with demyelination (*n* = 18; Figure 1g–i), and chronic CDV-DL with demyelination (*n* = 7; Figure 1j–l), according to previously published criteria [31,38]. Briefly, acute lesions showed either light-microscopically unchanged or mildly vacuolated parenchyma without evidence of loss of luxol fast blue (LFB)-positive white matter (Figure 1a,b). Subacute non-inflammatory lesions revealed vacuolation, mild to moderate gliosis, and mild loss of LFB-positive white matter (Figure 1d,e). Subacute inflammatory lesions exhibited mild, perivascular inflammatory infiltrates and moderate loss of LFB-positive white matter (Figure 1g,h). Chronic lesions revealed moderate to severe, perivascular inflammatory infiltrates and severe loss of LFB-positive white matter (Figure 1j,k). The control dogs did not show any inflammatory, degenerative or neoplastic lesions in the CNS (Figure 1m–o). The histological presumption of demyelination in the subacute and chronic subgroups as compared to controls and acute CDV-DL lesions was substantiated by demonstrating a moderately decreased percentage of 2′,3′-cyclic-nucleotide 3′-phosphodiesterase (CNPase)-positive oligodendrocytes as compared to the percentage of oligodendrocytes in the respective subgroups (Figure 2a). The histological presumption of gliosis in both subacute lesion subgroups as compared to controls and chronic CDV lesions was substantiated by demonstrating a slight increase of the percentage of glial fibrillary acidic protein (GFAP)-positive astrocytes (Figure 2b). An increased percentage of ionized calcium-binding adapter molecule 1 (Iba-1)-positive macrophages and microglia was evident in the subacute inflammatory and chronic subgroup as compared to controls and the acute and subacute non-inflammatory subgroup, respectively (Figure 2c).

### 2.2. Amount and Localization ROS-Induced Metabolites in CDV-DL

**Figure 3:**
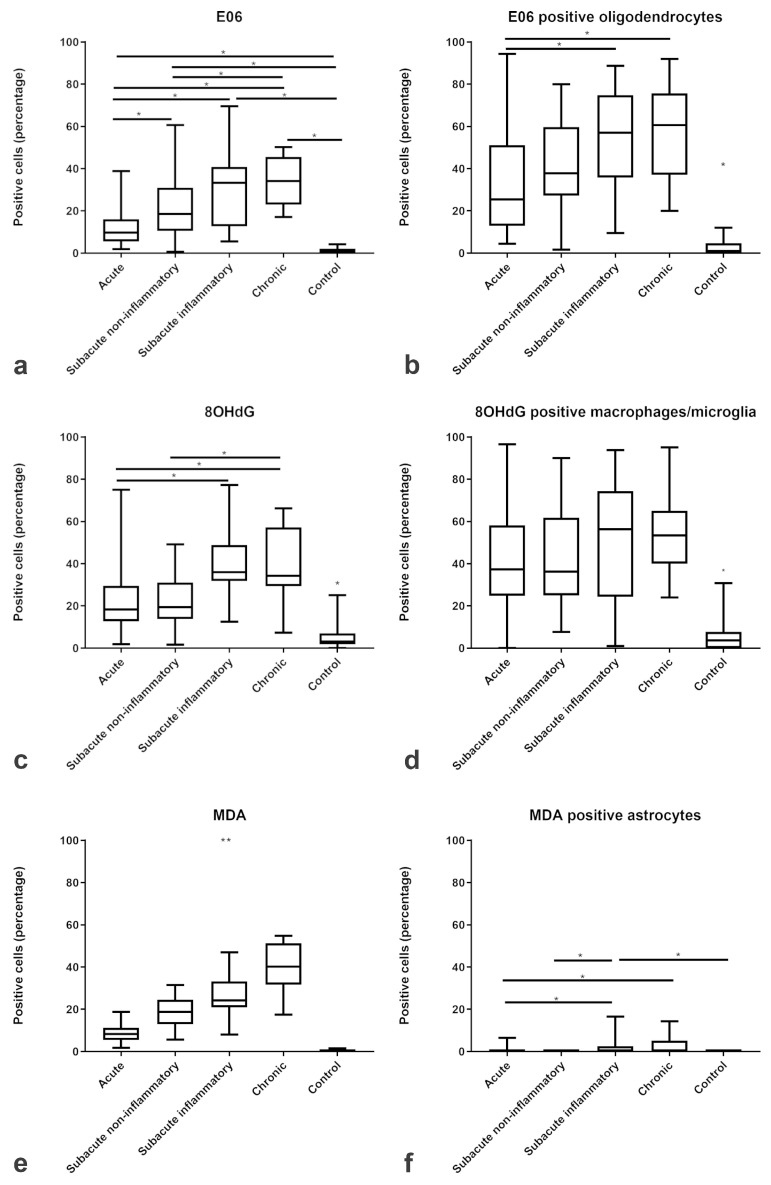
Relative changes in the proportions of cells immunopositive for ROS-products. The box plots show increasing percentages of positive cells for (**a**) oxidized phospholipids (clone E06), (**c**) 8-OHdG/8-OHG, and (**e**) MDA and the percentage of positive (**b**) oligodendrocytes, (**d**) macrophages/microglia and (**f**) astrocytes positive for ROS products in acute, subacute non-inflammatory, subacute inflammatory, and chronic CDV-DL lesions as compared to controls. Significant differences between the groups as revealed by the Kruskal–Wallis test with independent pairwise post-hoc Mann–Whitney U-test are marked with an asterisk (* *p* ≤ 0.05, ** *p* ≤ 0.005).

Immunofluorescence showed an increased percentage of intralesional cells immunoreactive for the three ROS-induced metabolites MDA, oxidized phospholipids (clone E06), and 8-OHdG/8-OHG with an increasing trend from acute to chronic lesions. The proportion of MDA-positive cells steadily increased from a median of 0% immunoreactive cells in controls to a median of 40% immunoreactive cells within chronic CDV lesions (Figure 3e). Comparably, oxidized phospholipids (clone E06) increased from a median of 1% in controls towards a median of 34% in chronic CDV lesions (Figure 3a), and 8-OHdG/8-OHG increased from a median of 3% in controls towards a median of 34% in chronic CDV lesions (Figure 3c).

**Figure 4:**
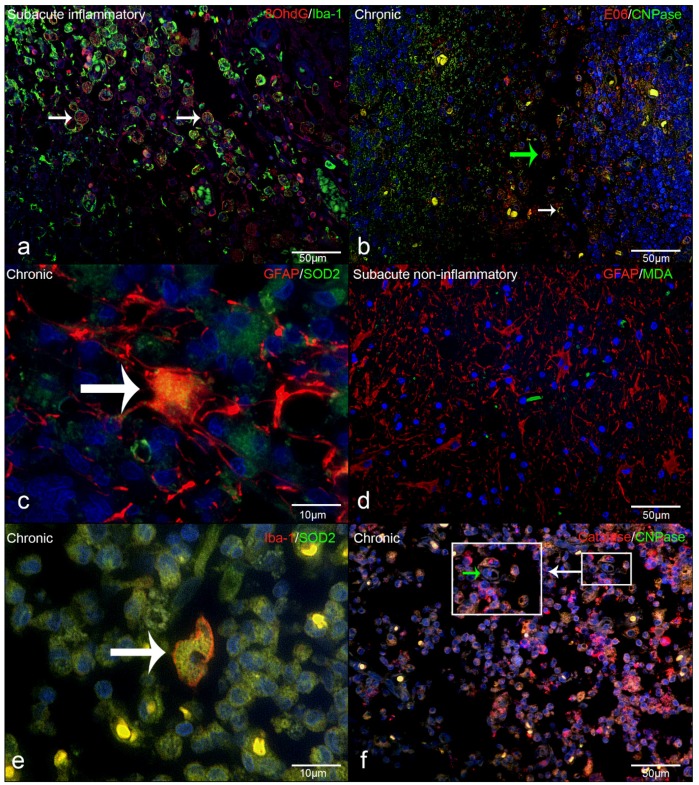
Immunofluorescence in CDV-DL lesions: (**a**) 8OHdG and Iba-1 as well as macrophages/microglia that are positive for 8OHdG (double labeling, yellow, arrow, (**b**) Oxidized phospholipids (clone E06) and CNPase as well as oligodendrocytes positive for oxidized phospholipids (double labeling, yellow, white arrow) as well as in cells with macrophage morphology (green arrow), (**c**) SOD2 and GFAP showing astrocytes positive for SOD2 (double labeling, yellow, arrow), (**d**) MDA and GFAP (red) double labeling (yellow) showing that astrocytes do not significant co-localize with MDA, (**e**) SOD2 and Iba-1 as well as macrophages/microglia positive for SOD2 (double labeling, yellow, arrow), (**f**) Catalase and CNPase, showing that oligodendrocytes can be positive for catalase (double labeling, yellow, green arrow within the insert).

Double immunofluorescence revealed that intralesional Iba-1-positive microglia/macrophages frequently demonstrated cytoplasmic co-localization with 8-OHdG/8-OHG (in the following text referred to as 8OHdG; Figure 3d). In detail, the percentage of intralesional microglia/macrophages harboring ROS-induced metabolites as revealed by Iba-1 and 8OHdG double immunofluorescence markedly increased from a median of 4% in controls to a median of 37% in acute, 36% in subacute non-inflammatory lesions, 56% in subacute inflammatory, and 53% in chronic lesions (Figure 3d). Co-localization of CNPase and oxidized phospholipid (clone E06-) immunoreactive granular masses was detectable in cells with morphology consistent with foamy macrophages (gitter cells; Figure 4b). Moreover, the percentage of intralesional oligodendrocytes harboring ROS-induced metabolites, as revealed by double-immunofluorescence for CNPase and oxidized phospholipids, markedly increased from a median of 1% in controls to a median of 25% in acute, 37% in subacute non-inflammatory lesions, 57% in subacute inflammatory lesions, and 61% in chronic lesions (Figure 3b). Double-immunofluorescence for MDA and GFAP revealed that astrocytes comprised no or only minimal amounts of ROS-induced metabolites (Figure 3f). Merely in subacute inflammatory lesions, there was a minor increase of intra-astrocytic MDA as compared to controls (Figure 3f).

**Figure 5:**
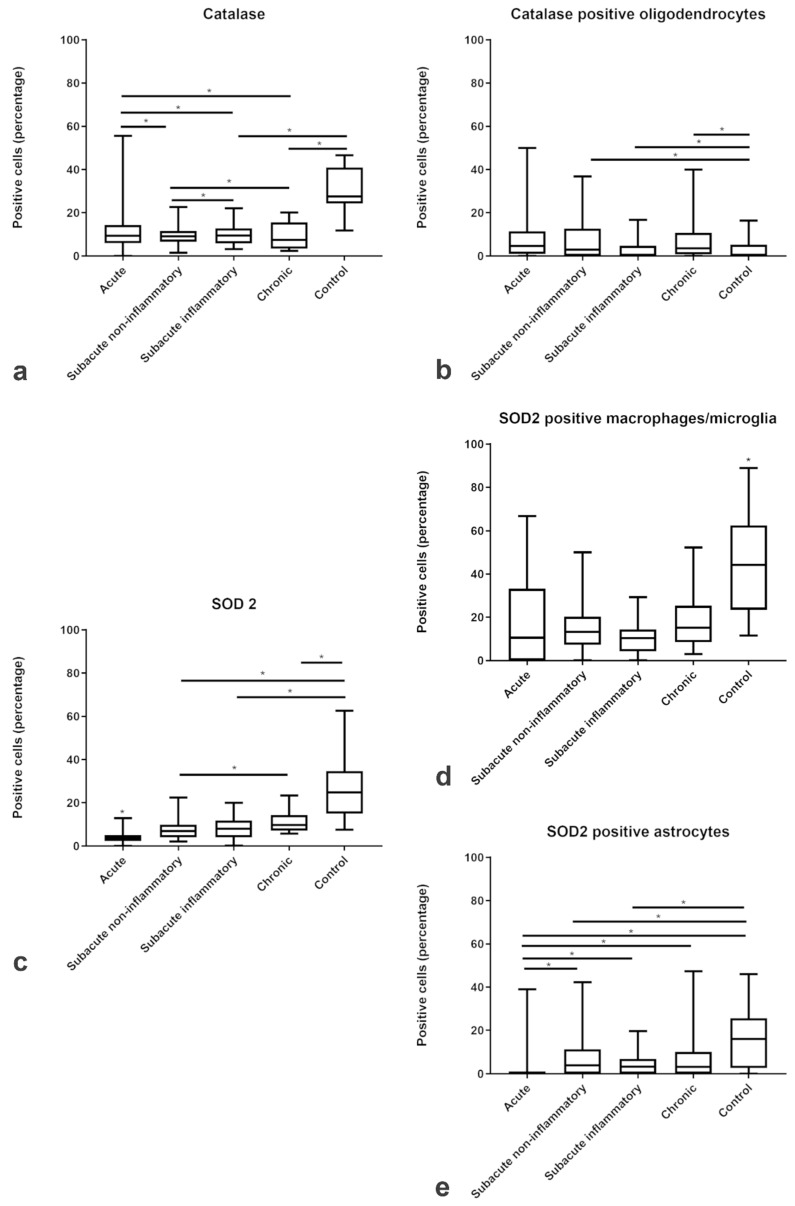
Relative changes in the proportions of cells immunopositive for antioxidative enzymes in CDV-DL. The box plots show the decreasing percentage of positive cells for antioxidant enzymes (catalase (**a**) and SOD2 (**c**)) and the percentage of positive (**b**) oligodendrocytes, (**d**) macrophages/microglia, and (**e**) astrocytes positive for antioxidant enzymes in acute, subacute non-inflammatory, subacute inflammatory, and chronic lesion compared to controls. Significant differences between the groups as revealed by the Kruskal–Wallis test with independent pairwise post-hoc Mann–Whitney U-test are marked with an asterisk (* *p* ≤ 0.05).

In contrast to the ROS metabolites, the expression of the antioxidant enzymes catalase and SOD2 was decreased within the lesions of all subgroups of CDV-DL as compared to controls (Figure 5a,c). Double-immunofluorescence revealed that oligodendrocytes expressed a low level of antioxidative enzymes, with only up to 5 % oligodendrocytes co-expressing CNPase and catalase (Figure 4f; Figure 5b). A more robust expression of antioxidative enzymes was observed in astrocytes and microglia/macrophages with up to 15% and 16% cells co-expressing GFAP and SOD2 (Figure 4c; Figure 5e) and Iba-1 and SOD2, respectively (Figure 4e; Figure 5d).

### 2.3. Lesion Profile in TMEV-DL

Within TMEV infected murine spinal cords there was mononuclear meningoleukomyelitis [37], beginning 14 dpi. The inflammatory changes increased towards 98 dpi, followed by a mild decline of the meningeal infiltrates towards 196 dpi. First demyelinated foci were detected in the ventrolateral funiculi at 42 dpi with a progressive increase of demyelination until 196 dpi. Mock-infected mice showed no demyelinating lesions. Based on morphologic alterations in CDV-DL lesions, the changes characterized by dilation of myelin sheaths with mild inflammatory infiltration at 14 dpi in TMEV-DL would roughly correspond to acute to subacute non-inflammatory lesions in CDV-DL. Analogously, subacute non-inflammatory lesions of CDV-DL most likely mimic TMEV-DL alterations at 42 dpi, while subacute inflammatory to chronic lesions in CDV-DL virtually resemble TMEV-DL lesions at 196 dpi [39]. The demyelination slightly starts at 14 dpi and a severe loss of myelin can be detected until 196 dpi [39]. Chronic CDV-DL lesions would thus be related to 245 dpi. However, it has to be emphasized that at 196 dpi a remyelination starts in TMEV-DL [39]. Statistical comparisons employing Mann–Whitney U-tests displayed a significantly higher degree of meningitis and perivascular inflammation in the white matter from 14 to 196 dpi, and demyelination from 42 to 196 dpi in TMEV infected compared to mock-infected mice, respectively [37].

### 2.4. Amount and Localization ROS-Induced Metabolites and Antioxidant Enzymes in TMEV-DL

**Figure 6:**
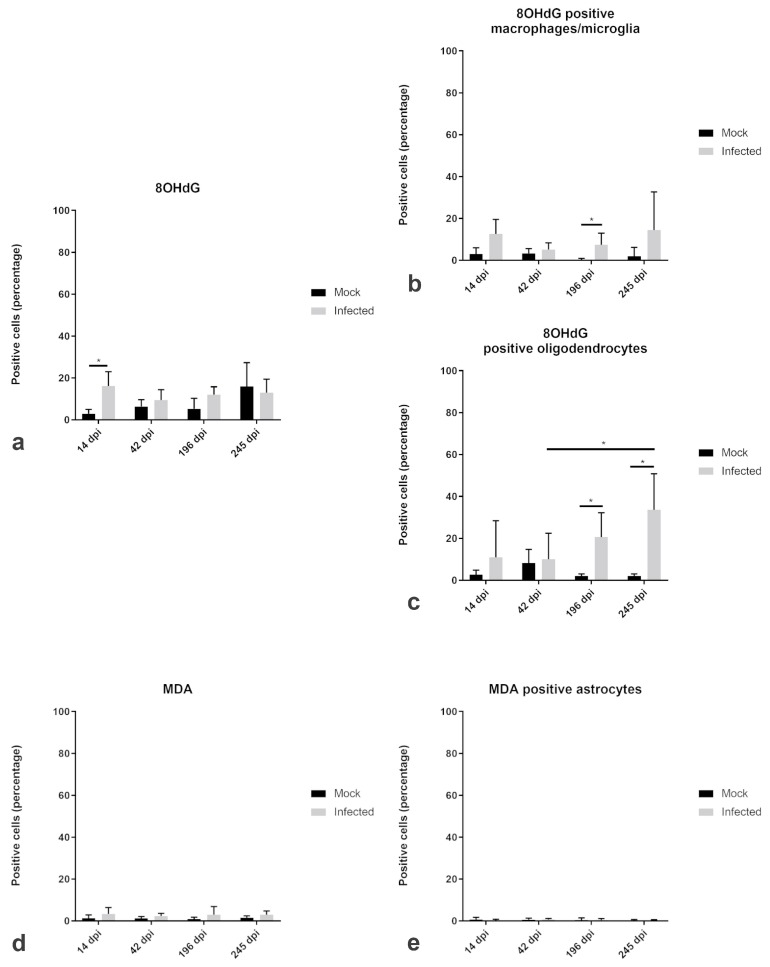
Relative changes in the proportions of cells immunopositive for reactive oxygen species (ROS) products and colocalization with astrocytes, oligodendrocytes, and microglia/macrophages in the white matter of spinal cord of TMEV-DL. The box plots show percentages of positive cells for ROS products (8-OHdG (**a**) and MDA (**d**)) and the percentage of double-positive (**b**) macrophages/microglia, (**c**) oligodendrocytes and (**e**) astrocytes at day 14, 42, 196, and 245 dpi compared to mock animals. Significant differences between the groups as revealed by the Kruskal–Wallis test with independent pairwise post-hoc Mann–Whitney U-test are marked with an asterisk (* *p* ≤ 0.05).

The percentage of cells immunopositive for 8OHdG was slightly increased in the infected spinal cord as compared to mock-infected animals. In detail, there was a significant increase of 8OHdG at 14 dpi in the infected animals (median 16.28%) in comparison to the mock animals (median 3.59%), respectively (Figure 6a). 8OHdG was predominantly expressed in macrophages and microglia (Figure 6b). Double-immunofluorescence revealed a significant increase of 8OHdG/Iba1-positive macrophages and microglia at 196 dpi (Figure 6b). Moreover, there was a significant increase of 8OHdG/CNPase-positive oligodendrocytes at 196 and 245 dpi, respectively (Figure 6c). Besides the amount of 8OHdG positive oligodendrocytes within the infected animals showed a significant increase between 42 dpi and 245 dpi within the white matter (Figure 6c).

In contrast to 8OHdG, MDA, which was only present in a very low percentage of cells, did not show any significant differences between TMEV-infected and mock infected animals (Figure 6d). There were only up to 10.95% of the cells immunopositive for MDA at day 196 dpi. Likewise, there were only few GFAP-positive astrocytes, co-localizing with MDA (Figure 6e).

**Figure 7:**
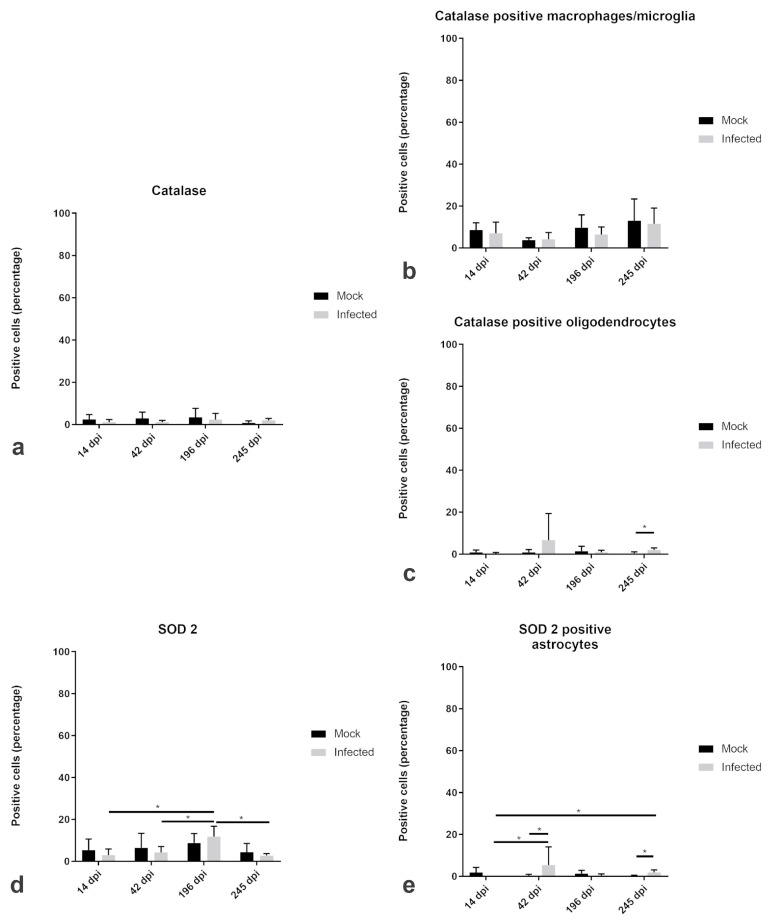
Antioxidant enzymes and colocalization with astrocytes, oligodendrocytes, and microglia/macrophages in the white matter of spinal cord of TMEV-infected mice. The box plots show percentage of positive cells for antioxidant enzymes ((**a**) catalase and (**d**) SOD2) and the percentage of positive (**b**) macrophages/microglia, (**c**) oligodendrocytes and (**e**) astrocytes at day 14, 42, 196, and 245 dpi compared to mock animals. Significant differences between the groups as revealed by the Kruskal–Wallis test with independent pairwise post-hoc Mann–Whitney U-test are marked with an asterisk (* *p* ≤ 0.05).

**Figure 8:**
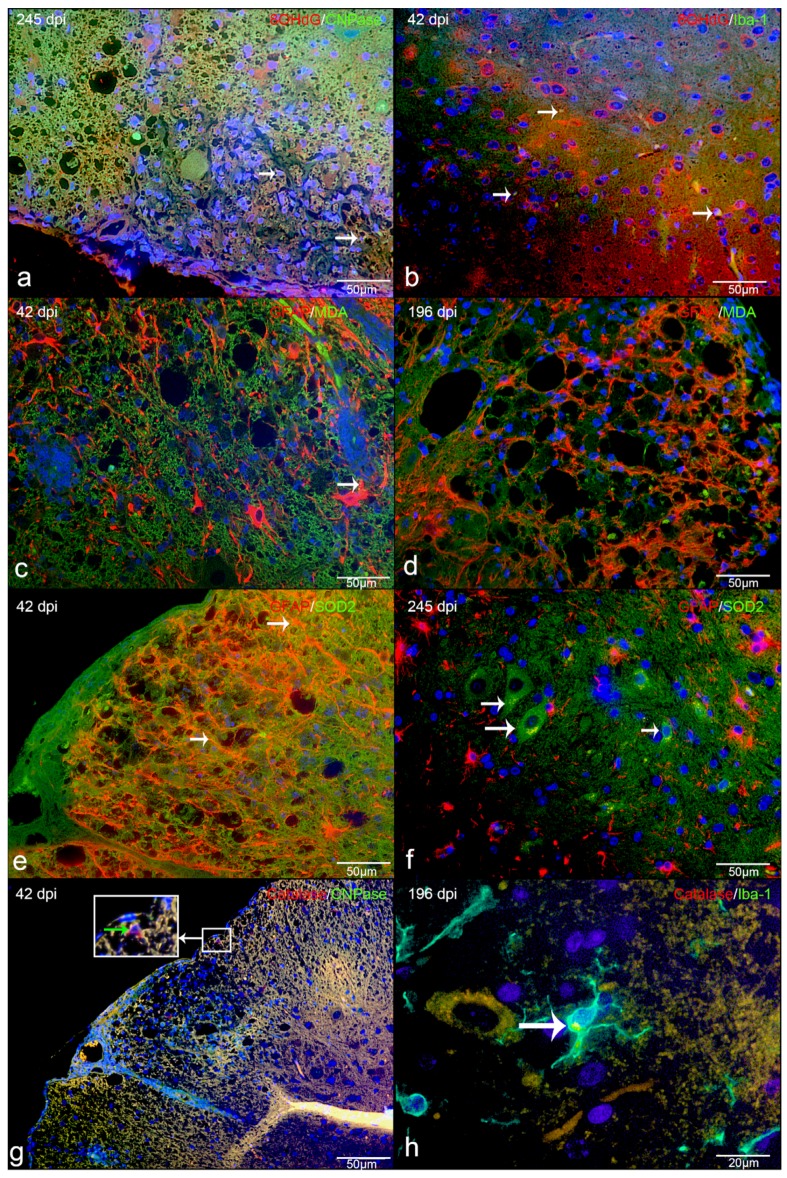
Immunofluorescence in TMEV-DL lesions: (**a**) 8OHdG and CNPase as well as oligodendrocytes positive for 8OHdG (double labeling, yellow, arrow) in the white matter of spinal cord of TMEV-DL, (**b**) 8OHdG and Iba1, showing that macrophages/microglia are positive for 8OHdG (double labeling, yellow, arrow) in TMEV-DL, (**c**) MDA and GFAP double labeling (yellow, arrow) in TMEV-DL, (**d**) MDA and GFAP, showing that astrocytes do not significant co-localize with MDA (double labeling, yellow) in TMEV-DL, (**e**) SOD2and GFAP as well as astrocytes positive for SOD2 (double labeling yellow, arrow) in TMEV-DL, (**f**) SOD2and GFAP double labeling (yellow) in TMEV-DL, some SOD2 positive cells share characteristics of neurons (arrow), (**g**) Catalase and CNPase, showing that oligodendrocytes can be positive for catalase (double labeling, yellow, green arrow within the insert) in TMEV-DL, (**h**) Catalase and Iba1 as well as macrophages/microglia positive for catalase (double labeling, yellow, arrow) in TMEV-DL.

The percentage of SOD2-positive cells was increased on 196 dpi (median 14.18%) in infected animals with a subsequent decrease to a median of 3.08% at 245 dpi (Figure 7d). Furthermore, infected animals did not show any astrocytes positive for SOD2 at 14 dpi. The percentage of catalase-positive cells did not differ between mock-infected animals and infected animals at any investigated time point. In fact, catalase was slightly increased in mock animal (up to 16.54%) than in TMEV- infected animal (up to 9.46%; Figure 7a). Catalase expression did not reveal significant differences between infected and non-infected animals. However, catalase positive macrophages and microglia increase from 14 dpi to 245 dpi (Figure 7b). In comparison, oligodendrocytes similarly co-localized with catalase but only in very low amounts. At 245 dpi, there was a significant difference between mock (median 0%) and infected (median 2.14%) animals (Figure 7c) in the white matter. Furthermore, SOD2 and catalase were present in cells that shared morphological characteristics with neurons (Figure 8f).

### 2.5. Transcriptional Activity of ROS-Generating and Detoxifying Pathways in CDV-DL

In order to substantiate the immunofluorescence data, a publicly available microarray data set of CDV-DL was filtered for a manually curated list of genes. KEGG-database and literature based genes that are related to ROS-production were subclassified into six functionally related groups: Mitochondrial respiratory chain complex I, III and IV (*n* = 50), NADPH oxidase enzyme system (*n* = 13), ROS detoxification system (*n* = 12), catabolic enzymes with oxidase activity (*n* = 3), pyruvate dehydrogenase complex (*n* = 5), and ROS toxification system (*n* = 4). The list corresponded to 87 genes in total. The fold changes and *p*-values for all of these 87 genes are displayed in Supplementary Table S4.

**Figure 9:**
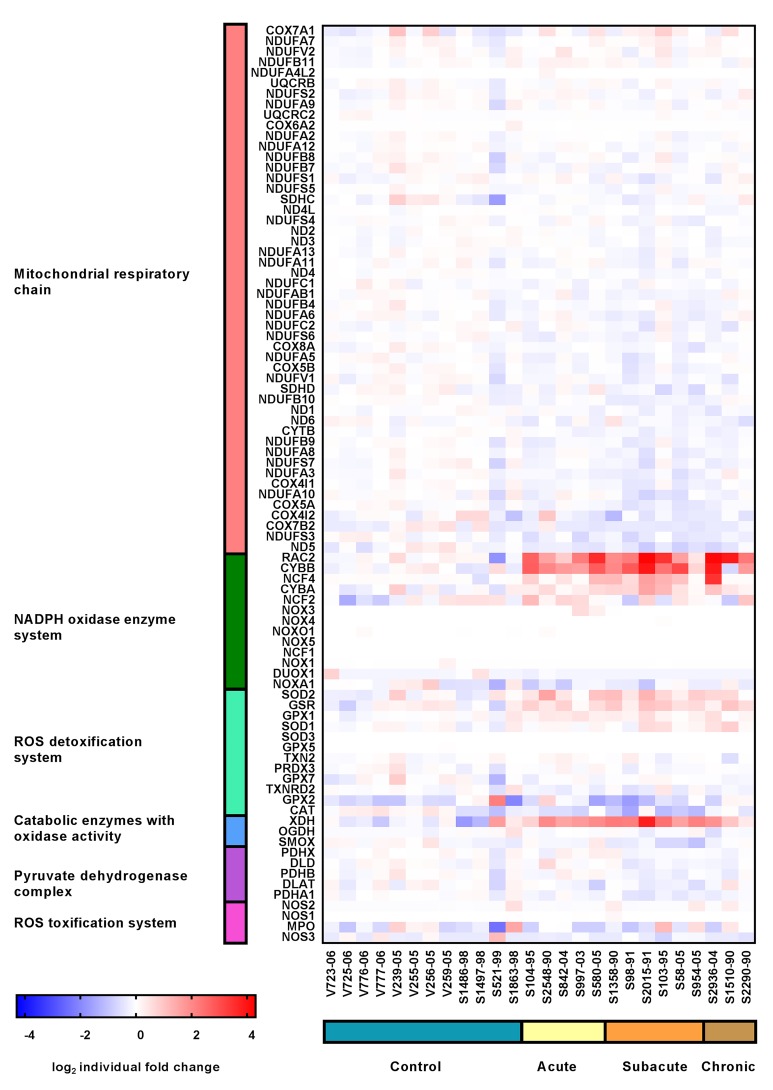
Expression profile of manually selected marker genes (rows) functionally related to mitochondrial respiratory chain (red), NADPH oxidase enzyme system (green), ROS detoxification system (mint), catabolic enzymes with oxidase activity (blue), pyruvate dehydrogenase complex (purple) and ROS toxification system (pink) in the cerebella (columns) of controls (blue), dogs affected by acute CDV-DL (sand), subacute CDV-DL with demyelination but without inflammation (ochre), and dogs affected by chronic CDV-DL with demyelination and with inflammation (brown). Note that the most prominent changes in gene expression are upregulations of genes of the NADPH oxidase enzyme system affecting all subgroups of CDV-DL. The heatmap displays the log2-transformed individual fold changes relative to the mean expression of the controls indicated by a color scale ranging from −4 (16-fold downregulation) in blue to 4 (16-fold upregulation) in red.

Transcriptional analysis revealed a downregulation of genes related to mitochondrial respiratory chain in all groups of CDV-infected dogs. This downregulation was obvious already in the acute lesions and reached a maximum of 34% differentially expressed genes at the onset of demyelination in subacute non-inflammatory CDV-DL with demyelination (Table 1). The gene with the highest fold change in all 3 groups was NADH dehydrogenase subunit 5 (ND5) which was mildly downregulated (–1.27, –1.41, and –1.31-fold) in all subgroups of CDV-DL as compared to controls, respectively (Figure 9).

In contrast to the genes of the mitochondrial respiratory chain complex, 30.8% of the genes of the NADPH oxidase enzyme system revealed an upregulation in CDV-infected dogs as compared to controls (Table 1). Maximum fold changes reached 4.78 and 11.44-fold upregulation of ras-related C3 botulinum toxin substrate 2 (rho family, small GTP binding protein Rac2; RAC2) in acute CDV-DL and chronic CDV-DL with demyelination, and a 5.71-fold upregulation of cytochrome b-245, beta polypeptide (CYBB) in subacute non-inflammatory CDV-DL with demyelination as compared to controls, respectively (Figure 9).

25% of genes of the ROS detoxification system revealed an upregulation, and 8.33% showed a downregulation in CDV-infected dogs as compared to controls (Table 1). Maximum fold changes were a –1.77-fold downregulation for catalase and a 1.75-fold upregulation for superoxide dismutase 2 (SOD2) in subacute non-inflammatory CDV-DL with demyelination (Figure 9).

33.33% of the genes of the catabolic enzymes with oxidase activity revealed an upregulation in acute CDV-DL, whereas in subacute non-inflammatory CDV-DL with demyelination and chronic CDV-DL with demyelination subgroups there were 33.33% up- and 33.33% downregulated genes (Table 1). The maximum fold change was a 6.24-fold upregulation of xanthine dehydrogenase (XDH) in subacute non-inflammatory CDV leukoencephalitis with demyelination (Figure 9).

20% of genes of the pyruvate dehydrogenase complex revealed a downregulation in subacute CDV leukoencephalitis with demyelination but without inflammation (Table 1).

Genes of the ROS toxification system did not show any changes.

### 2.6. Transcriptional Activity of ROS-Generating and Detoxifying Pathways in TMEV-DL

Analogous to CDV-DL a publicly available microarray data set of mice affected by TMEV-DL [37] was investigated for changes in the above-mentioned gene sets. Similar to CDV-DL, these genes were sub divided into six functionally related groups: Mitochondrial respiratory chain I, III, and IV (*n* = 55), NADPH oxidase enzyme system (*n* = 9), catabolic enzymes with oxidase activity (*n* = 3), pyruvate dehydrogenase complex (*n* = 5) and ROS toxification system (*n* = 4), and ROS detoxification system (*n* = 17), corresponding to a total list of 93 genes. The fold changes and *p*-values for all of these 93 genes are displayed in Supplementary Table S4.

In general, the transcriptional analysis showed most prominent upregulation of genes of the groups NADPH oxidase enzyme system, ROS detoxification system, and catabolic enzymes with oxidase activity. Downregulation of genes were present in the groups of mitochondrial respiratory chain I, III, and IV, pyruvate dehydrogenase complex and ROS toxification system.

**Figure 10:**
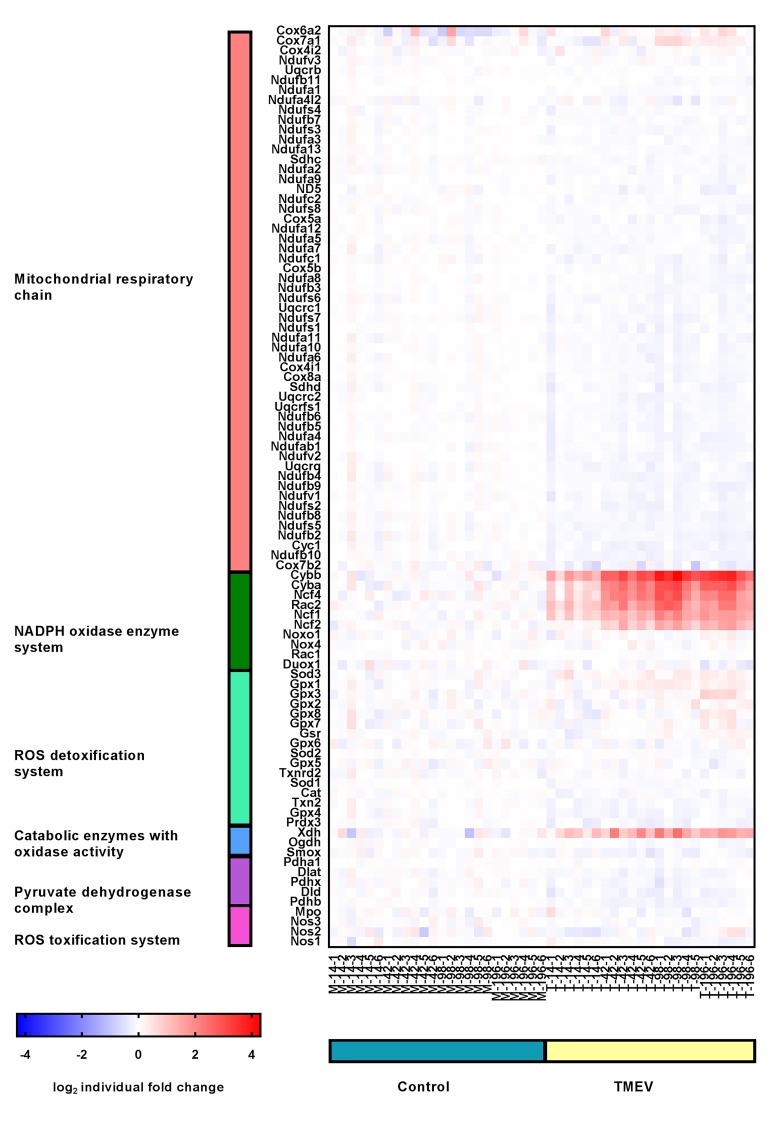
Expression profile of manually selected marker genes (rows) functionally related to mitochondrial respiratory chain (red), NADPH oxidase enzyme system (green), ROS detoxification system (mint), catabolic enzymes with oxidase activity (blue), pyruvate dehydrogenase complex (purple) and ROS toxification system (pink) in the spinal cord (columns) of controls (blue) and TMEV infected mice (brown). Note that the most prominent changes in gene expression are upregulations of genes of the NADPH oxidase enzyme system affecting TMEV infected mice. The heatmap displays the log2-transformed individual fold changes relative to the mean expression of the controls indicated by a color scale ranging from −4 (16-fold downregulation) in blue to 4 (16-fold upregulation) in red.

Similar to CDV-DL, transcriptional analysis revealed an upregulation (up to 66.7%, Table 2) of genes related to NADPH oxidase enzyme system in TMEV infected mice. Maximum fold changes were present in cytochrome b-245, beta polypeptide (CYBB) at day 42 pi (6.94-), day 98 pi (12.14-), and day 196 pi (9.41-fold) as compared to mock infected animals, respectively (Table 2, Figure 10).

In contrast to the NADPH oxidase enzyme system, genes related to the ROS toxification system as well as the mitochondrial respiratory chain complex I, III, and IV showed downregulation in the infected animals compared to the mock-infected animals. Most of the genes in the group of mitochondrial respiratory chain complex I, III, and IV were downregulated at day 196 pi (65.45%, Table 2). Maximum fold changes were −1.21 for NADH dehydrogenase (ubiquinone) 1 alpha subcomplex, 4-like 2 at 98 dpi.

Downregulation of genes of the group ROS toxification system was present at day 98 and 196 pi, respectively (25%, Table 2). A −1.14-fold downregulation at day 196 pi of nitric oxide synthase 1, neuronal, represent the maximum fold change in this group.

66.7% of the genes of the group catabolic enzymes with oxidase activity revealed an upregulation with maximum fold changes reaching 2.88 at day 196 pi in the infected animals. The other half of genes in this group showed downregulation at day 42 pi with a minimum fold change of −1.18 (Table 2).

23.53% of genes of the ROS detoxification system revealed an upregulation at day 196 pi. 17.65% of genes from the same group showed a downregulation at day 98 and 196 pi, respectively (Table 2). Maximum fold changes were a 1.23-fold upregulation of glutathione peroxidase 1 at day 98 pi. The maximum downregulated fold change (–1.10) showed catalase at day 196 pi.

60% of the genes from the pyruvate dehydrogenase complex were downregulated in comparison to the mock-infected animals (Table 2). The maximum fold change was reached by pyruvate dehydrogenase complex, component X with a −1.18-fold downregulation at day 98 pi.

### 2.7. Intersections Between Differentially Expressed Genes in CDV-DL and TMEV-DL

**Figure 11:**
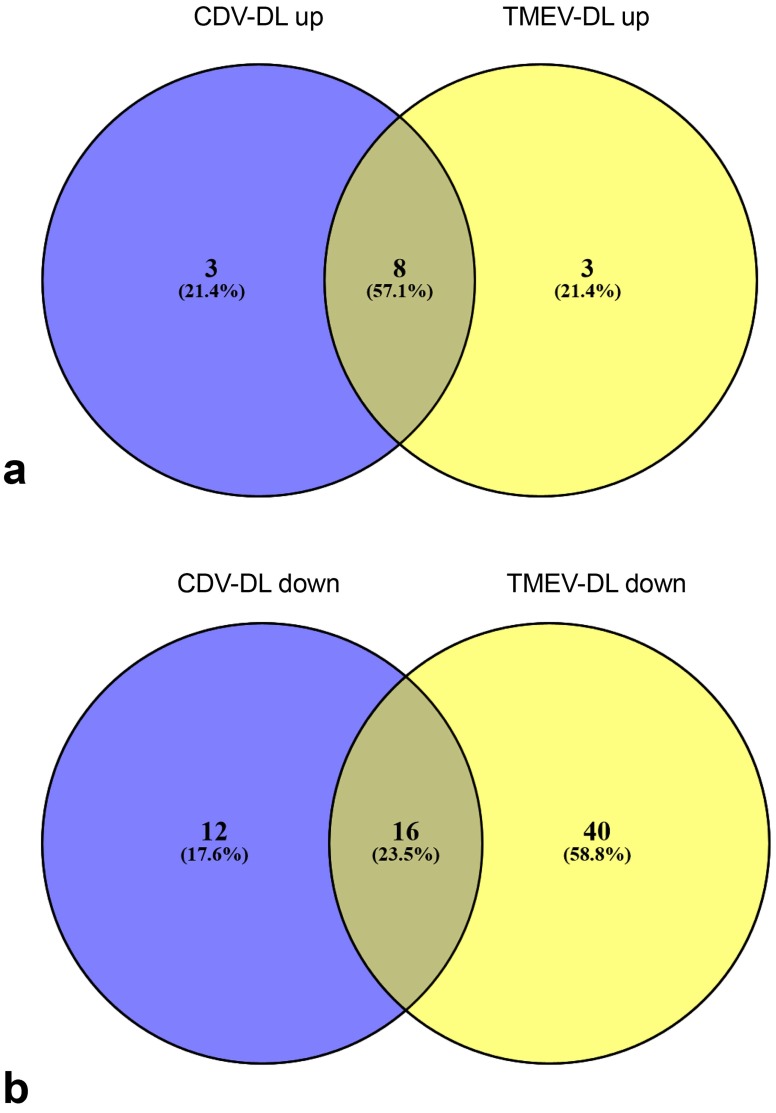
Venn diagrams comparing the differentially expressed genes (DEGs) of a manually generated list of genes implicated in the generation and detoxification of ROS-metabolites in CDV DL and TMEV infected mice: (**a**) the Venn diagram display the number and intersections of upregulated DEGs in CDV leukoencephalitis and TMEV infected mice, respectively. The three genes included exclusively in CDV-DL were: UQCRC2, SOD1, and SOD2. The three genes included exclusively in “TMEV-DL up” were GPX3, NCF1, and SOD3. (**b**) the Venn diagram display the number and intersections of downregulated DEGs in CDV leukoencephalitis and TMEV infected mice, respectively.

The differentially expressed probesets of the manually selected genes, implicated in ROS generation and detoxification, respectively, of CDV-DL (*n* = 87) and TME (*n* = 93) were compared between the two models using Venn diagrams (Figure 11). A total number of 11 genes was upregulated in at least one group contrast as compared to controls in both CDV-DL and TME. Interestingly, 8 of these genes were upregulated in both and thus shared between the animal models (XDH, CYBA, CYBB, NCF2, NCF4, RAC2, GPX1, GSR), while 3 genes were each exclusively upregulated in CDV-DL and TME, respectively (Figure 11).

A total number of 28 out of 87 genes were downregulated in CDV-DL and 56 out of 93 genes were downregulated in TME. Both animal models shared an intersection of 16 genes, which were similarly downregulated in both diseases (SMOX, ND5, NDUFA10, NDUFA3, NDUFA6, NDUFA8, NDUFAB1, NDUFB10, NDUFB9, NDUFS6, CAT, PDHA1, COX4I1, COX5A, COX7B2, COX8A). The 12 genes exclusively downregulated in CDV-DL were CYTB, ND1, ND3, ND4, ND4L, ND6, NDUFS3, GPX2, PDHB, OGDH, COX4I2, and TXNRD2. The 16 genes exclusively downregulated in TMEV-DL were SMOX, ND5, NDUFA10, NDUFA3, NDUFA6, NDUFA8, NDUFAB1, NDUFB10, NDUFB9, NDUFS6, CAT, PDHA1, COX4I1, COX5A, COX7B2, and COX8A. The 40 downregulated genes shared by both diseases were CYC1, DUOX1, GPX4, NDUFA1, NDUFA11, NDUFA12, NDUFA13, NDUFA2, NDUFA4, NDUFA4L2, NDUFA5, NDUFA7, NDUFA9, NDUFB11, NDUFB2, NDUFB3, NDUFB4, NDUFB5, NDUFB6, NDUFB8, NDUFC2, NDUFS1, NDUFS2, NDUFS5, NDUFS7, NDUFS8, NDUFV1, NDUFV2, NOS1, PRDX3, SOD1, UQCRC1, UQCRC2, UQCRFS1, UQCRQ, DLAT, PDHX, COX5B, COX7A1, and TXN2 (fold changes and *p*-values can be seen in Supplementary Table S4).

## 3. Discussion

Within active MS lesions immunohistochemistry revealed products of ROS reaction (MDA, oxidized phospholipids and 8OHdG) within oligodendrocytes, astrocytes, neurons, myelin, and macrophages and DNA and lipid damage has been demonstrated to be associated with demyelination [11]. Furthermore, a microarray study showed an upregulation of genes that are related to increased ROS production [40]. Increased amounts of ROS have also been described in traumatic brain and spinal cord injuries as well as Alzheimer’s disease [41]. These observations lead to the hypothesis that ROS generation might also represent a key feature in the pathogenesis of CDV-DL and TMEV-DL, both representing virally induced animal models for demyelinating CNS disease. The present study demonstrates for the first time that ROS play a substantial albeit variable role in CDV-DL and TMEV-DL. While TMEV-infection is a well-established experimental mouse model of demyelinating disease [42,43], CDV-DL represents one of the relatively rare naturally occurring models for human demyelinating CNS conditions such as MS [44]. Though ROS have been previously demonstrated to play a pivotal role in the pathogenesis of demyelinating disease such as MS and its animal models [45], their role in CDV-DL and TMEV-DL has so far not been investigated. Both CDV-DL and MS show non-inflammatory acute demyelination followed by an inflammatory infiltrate composed of macrophages and lymphocytes as well as a subacute to chronic progressive demyelination [23]. Similarly, TMEV-induced demyelinating lesions in mice share pathological similarities with MS lesions, and the immune response of infected mice appears to play an important role in the pathogenesis of demyelination [46]. The present study was performed in order to evaluate the amount and localization of ROS induced metabolites and detoxifying enzymes in CDV-DL and TMEV-DL in situ, and to comparatively evaluate the transcriptional activity of important ROS generating as well as detoxifying pathways in publicly available microarray datasets of CDV-DL and TMEV-DL.

The main resources of superoxide production are NADH dehydrogenase and ubisemiquinone, which represent the complex I and III of the electron transport chain, respectively [2,3,47]. In the microarray study, genes of the NADPH oxidase enzyme system were upregulated, possibly indicating that NAPDH oxidase is a major source of the immunohistochemically detected ROS products in CDV-DL. In CDV-DL up to 56.25% of the intralesional microglia/macrophages were positive for the oxidative DNA/RNA damage marker 8OHdG, as compared to only up to 3.63% in control dogs.

Microglia/macrophages are known to represent a major source of ROS in response to infection or pro-oxidant cytokine stimulation such as interleukin-1 (IL-1) and tumor necrosis factor (TNF) [48]. These pro-inflammatory mediators have previously been demonstrated to be increased in CDV-DL lesions [28,49]. NADPH oxidase (NOX2) itself only represents one enzyme of the NOX enzymes [50]. The NOX enzymes are a group of enzymes that are composed of NOX1, NOX2, NOX3; NOX4, NOX5, DUOX1, and DUOX2. ROS are produced by NADPH oxidase during respiratory burst, which is for instance used by phagocytes to kill foreign organisms [47,50]. In addition, myeloperoxidase in macrophages and microglia contribute to generation of ROS during phagocytosis [51]. Rac GTPases are key regulator proteins of the NOX enzymes [50] and therefore regulate the production of superoxide [52]. Interestingly, the microarray data in the present study revealed an increase of ras-related C3 botulinum toxin substrate 2. This molecule has several functions in morphology changes, cell adhesion, and migration [53] and belongs to the GTP-binding proteins [54,55]. Therefore, this result may indicate an increased activation of NOX enzymes by Rac and consequently an increased ROS production by inflammatory cells during the time course of CDV-DL lesions.

In neurodegenerative diseases, such as Alzheimer’s disease, Friedreich ataxia, Parkinson Disease and MS, high levels of iron may be part in the pathogenesis [10,56,57]. Iron in combination with oxidative stress leads to Fenton reaction and increased occurrence of ROS-induced damage [10,56,57]. Oligodendroglia are highly susceptible to ROS because of their high iron content [58]. Notably, up to 94.29% of oligodendrocytes co-localized with the oxidized lipid marker E06 in CDV-DL. This is highly similar to MS in humans [45]. Within the white and grey matter of human brains suffering from active MS lesions, oxidized lipids have been shown to be present in oligodendrocytes, astrocytes, and neurons [11,45]. Both iron and oxidized phospholipids have been demonstrated in oligodendrocytes of human brains suffering from MS [10]. Moreover, there is immunoreactivity for MDA and oxidized phospholipids as well as 8OHdG in the cytoplasm of oligodendrocytes as well as within some astrocytes in paraffin embedded brain tissue from MS patients [11]. Furthermore, increased amounts of oxidized lipids correlated with infiltration of microglia/macrophages in MS by immunohistochemistry [11,40,45]. It is known that one likely source of ROS in demyelinating diseases are inflammatory cells such as macrophages and microglia [40,47]. In the present study upon CDV-DL, there were similarly higher numbers of macrophages/microglia in parallel to increased ROS product immunoreactivity, thus mirroring results from brain tissue of MS patients. Moreover, the co-localization of this ROS metabolite product and oligodendrocytes may indicate ROS-induced damage of oligodendrocytes in CDV-DL, which may be related to the pathogenesis of demyelination in this disease [4].

In contrast to oligodendrocytes and microglia/macrophages, only up to 14.29% of the intralesional astrocytes were positive for MDA in CDV-DL. It has previously been shown that astrocytes exhibit a higher resistance to free iron than neurons and vascular endothelial cells within the brain [56], thus indicating a relative resistance of astrocytes to ROS-induced damage, which is in concordance to the present study.

Parallel evaluation of publicly available microarray data of CDV-DL lesions demonstrated several genes of ROS production being upregulated. Specifically, upregulation of NADPH enzymes was a prominent finding in the evaluation of transcriptome data of CDV-DL lesions. It was previously demonstrated that major histocompatibility complex class II (MHCII), which indicates activated microglia, is upregulated in CDV-DL lesions [26]. Interestingly, activated microglia are known to be potent producers of ROS by NADPH oxidase [47,50]. NOX-enzymes appear not to be expressed in oligodendrocytes themselves [50]. The parallel observation of increased ROS products in Iba-1 positive microglia/macrophages and upregulation of NADPH oxidase on the transcriptome level indicates ROS production by microglia/macrophages as a pivotal factor in the pathogenesis of CDV-induced demyelinating CNS disease and thus also substantiates CDV-DL as a naturally occurring animal model for human demyelinating disease such as MS. Moreover, high NADPH/NAD^+^ ratios and a high proton motive force in mitochondria also leads to mitochondrial damage [59]. Mitochondrial destruction has previously been implicated in the pathogenesis of MS [1,51,60,61].

To avoid superoxide-induced damage, mitochondria contain SOD2 [62]. Previous studies may indicate a higher susceptibility of oligodendrocytes to ROS due to a relative lack of detoxifying enzymes in this glial cell type [19,63]. Moreover, the high lipid content of myelin contributes to the fact that oligodendrocytes and their precursor cells are extremely vulnerable to ROS induced damage [4,13,14,15]. Catalase can delay demyelination in experimental optical neuritis in guinea pigs, while SOD does not appear to have any effects upon the extent of demyelination [64]. In the present study, the antioxidant enzymes catalase and SOD2 were significantly decreased within the lesions of all subgroups of CDV-DL as compared to controls with very low numbers of oligodendrocytes co-expressing CNPase and catalase. As compared to oligodendrocytes, astrocytes and microglia/macrophages showed a more robust expression of these markers. In contrast to the reduced amount of protein as revealed by immunofluorescence, microarray data of CDV-DL revealed a mild increase of SOD2 mRNA expression. This currently unexplained discrepancy may be due to an increased post-translational turnover of the protein. Catalase transcription was, similar to the protein level, mildly downregulated. These results underline that ROS-induced damage to the poorly protected oligodendrocytes is an important pathogenetic feature leading to demyelination in CDV-DL. Similar findings were interestingly demonstrated in MS lesions [65]. In contrast to CDV-DL, the presented data of TMEV-DL revealed robust differences on the protein level. Though, analogously to CDV-DL, ROS metabolite products co-localized with macrophages, microglia as well as oligodendrocytes, the amount of positive cells was substantially lower in TMEV-DL as compared to CDV-DL. Interestingly, it has been previously been demonstrated that there is a difference regarding the role of ROS in MS and mouse models for demyelinating disease such as experimental autoimmune encephalomyelitis (EAE) and mouse hepatitis virus infection [11,45]. In contrast to CDV-DL, the amount of 8OHdG was exclusively increased at 14 dpi in comparison to the controls in TMEV-DL. Interestingly, and similarly in stark contrast to CDV-DL, MDA did neither show significant changes during the time course of TME induced demyelination. Catalase and SOD2 showed the highest amount in the mock infected animals. Interestingly only low amounts of SOD2 were present within astrocytes and astrocytes generally showed only low amounts of ROS products. Low amounts of catalase were also present within oligodendrocytes, which may indicate the low antioxidant defense of these cells and the vulnerability to ROS.

Finally, though differing in the extent, both TMEV-DL and CDV-DL showed presence of ROS within the lesions. Though the presented data do not allow a final conclusion on the effects of ROS during the demyelination process, the similarity to MS may suggest that ROS are indeed causally involved in the pathogenesis of demyelination in both models. The relative lower amount of ROS in TMEV-DL may further indicate that at least based on the role of ROS CDV-DL might represent a more appropriate model for the role of ROS during demyelinating disease.

The present study certainly has limitations. First, we compared a naturally occurring disease in dogs with an experimental mouse model. The overall higher contribution of ROS to the disease process in dogs may thus have also been influenced by environmental factors and a more heterogeneous composition of the naturally induced lesions. Another limitation of the present study is represented by the fact that two different compartments of the CNS have been investigated in both models. It cannot be ruled out that ROS play differing roles in the cerebellum as compared to the spinal cord and this may have affected the presented results. Moreover, besides the disease entity the species itself may have an influence upon the general contribution of ROS to the disease process.

## 4. Materials and Methods

### 4.1. Ethics Statement

This study was conducted in accordance with the German Animal Welfare Act. The authors confirm that the CDV-infected dogs included in the present study were not infected or sacrificed for the purpose of this retrospective pathological case-control study. Therefore, our experiments using archival formalin-fixed, paraffin-embedded dog tissues are not an animal experiment since all animals were dead at the time of submission for necropsy in order to investigate the causes of death and disease. These tissue samples were all collected by one of the authors (WB) during his work at the diagnostic pathology services of the Department of Pathology, University of Veterinary Medicine Hannover, and the Institute of Veterinary Pathology, Justus-Liebig-University Giessen. Some of the animals were used in previous publications [31,66].

The TMEV-infection experiments using mice were authorized by the local authorities (Regierungspräsidium Hannover, Germany, permission number: 33–42502-05/963 (30 May 2005) and 33-42502-04-07/1292 (14 June 2007)) and are described in more detail in previous publications [67].

### 4.2. Experimental Design

Breed, age, and sex of the 16 CDV-infected and 8 negative control dogs are presented in Supplementary Table S1. All CDV-infected dogs were naturally infected and were diagnosed as CDV-DL-positive in the context of diagnostic pathological services. The current case-control study was restricted to the cerebellar specimens of these dogs. These showed focal or multifocal lesions of single or multiple subtypes of CDV leukoencephalitis within each individual dog which were subdivided based on histopathological findings according to previous descriptions [26,66]. The control dogs did not show any morphological evidence of CNS disease and were immunohistologically negative for CDV antigen.

Five-week-old female SJL/JHanHsd-mice (Harlan Winkelmann, Borchen, Germany; transcriptome analysis) or SJL/JCrl-mice (Charles River Laboratories, Sulzfeld, Germany; immunohistology experiments) were inoculated into the right cerebral hemisphere with 1.6 × 10^6^ or 4.6 × 10^7^ plaque forming units per mouse of the BeAn-strain of TMEV or cell culture medium (mock-infection), respectively, in two independent experiments, as previously described [67].

### 4.3. Histology and Immunofluorescence

Two to three micrometer thin serial sections were cut from formalin-fixed, paraffin-embedded CNS tissue using a rotary microtome (Leica, Nußloch, Germany), mounted on Menzel Gläser Superfrost ^®^ Plus slides (Thermo Fisher Scientific Inc, Gerhard Menzel B.V. & Co KG, Braunschweig, Germany.), and stained with hematoxylin and eosin (HE) and luxol fast blue-cresylechtviolet (LFB-CV).

For evaluation of ROS-products and antioxidant enzymes immunofluorescence was performed. A representative panel of various antibody combinations was made to evaluate ROS induced metabolites from either lipid oxidation (oxidized phospholipids or MDA) or damaged DNA or RNA (8OHdG). According to the literature, oxidized lipids have previously detected within the cytoplasm of astrocytes in MS lesions of human brains [11,45]. Due to technical reasons, for example the need to combine two primary antibodies from two different species limited the combination of antibodies. The dilution of the antibodies and the pretreatments are listed in Supplementary Table S2. Deparaffination of the paraffin embedded tissue was performed in Roticlear (Carl Roth GmbH, Karlsruhe, Germany), isopropanol and 96% ethanol. Afterwards slides were washed three times in phosphate buffered saline (PBS), followed by pretreatment (Supplementary Table S2). Slides were either placed in 10 mM Na-citrate buffer pH 6.0, for 20 min in a microwave oven (800 W) or placed in distilled water with proteinase K (Proteinase K, recombinant PCR Grade, Roche Diagnostics GmbH, Mannheim, Germany), for 15 min. For blocking unspecific binding, serum from the host species of the secondary antibody was used. Sections were incubated with primary antibodies for 90 min at room temperature (primary antibodies, see Supplementary Table S2). Negative control sections were incubated with equally diluted normal rabbit serum (Sigma-Aldrich, R9759), a mouse IgG1 anti-isotype antibody (Millipore, CBL600), or non-immunized goat serum instead of the primary antibodies. As a positive control for ROS metabolites, a canine spinal cord with a fibrocartilaginous embolus (FCE) was used [68]. The positive control of CDV-antigen was a cerebellum of a confirmed CDV-positive dog. Subsequently, sections were incubated with fluorescent goat-anti–mouse, goat-anti-rabbit, donkey-anti goat, or donkey-anti-rabbit antibodies (secondary antibodies, see Supplementary Table S3) for one hour at room temperature. Nuclei were stained with bisbenzimide (Hoechst 33258, 0.01 % in methanol, Sigma-Aldrich, Taufkirchen, Germany) diluted 1:100 in distilled water for 10 min. The slides were mounted using Dako Fluorescence Mounting Medium (DakoCytomation, Hamburg, Germany) [69].

Immunofluorescence sections were evaluated with a fluorescence microscope (Keyence BZ-9000E, Keyence, Mechelen, Belgium) equipped with the following filters: (1) A hard-coated band pass filter for bisbezimide (DAPI, emission 447/60 nm, excitation 377/55 nm), (2) a hard-coated band pass filter for Cy2 (TexasRed, emission 624/40 nm, excitation 562/40 nm), and (3) a hard-coated band pass filter for Cy3 (GFP, emission 520/35nm, excitation 473/30 nm), respectively, as described [70]. The sections were scanned with the CFI Plan Apo 10x ʎ NAO.45 WD 4.0 objective and analyzed by using the BZ-II-analyzer software CBZ-HZAE (Keyence, Mechelen, Belgium).

The assessment of the immunofluorescent slides was based on manually defined and outlined lesioned areas in CDV-DL and TMEV-DL. All cells within these predefined lesions were counted manually. The number of bisbenzimide-positive nuclei corresponds to 100% of cells. Subsequently, the numbers of cells immunoreactive for the different ROS metabolites (oxidized phospholipids, MDA, 8OHdG/8OHG), ROS detoxifying enzyme (SOD, catalase) and cell-type specific markers (Iba-1, GFAP, CNPase) were counted in the same areas. The number of the total cell count was variable between the different lesion types. It ranged from a minimum of 55 cells per lesion to a maximum of 982 cells per lesion to estimate the proportion of positive cells. The percentage of positive cells per cell type as assessed by immunofluorescence was compared for significant differences between the groups using a non-parametric Kruskal–Wallis test with post-hoc independent pairwise Mann–Whitney U-tests (IBM SPSS Statistics, Version 24; IBM Corporation, Armonk, NY, USA). *p*-values ≤ 0.05 were defined as significant. Box-and-whisker plots were created using GrapPad Prism, Version 7.02 (Graph-Pad Software, La Jolla, CA, USA).

### 4.4. Microarray Analysis

For the transcriptome analysis of CDV-DL, RNA was isolated from snap frozen cerebellar specimens using the Rneasy Lipid Tissue Mini Kit (Qiagen, Hilden, Germany), amplified and labelled employing the 3′IVT express kit (Affymetrix, Santa Clara, USA), and hybridized to GeneChip canine genome 2.0 arrays (Affymetrix, Santa Clara, USA) as previously described [31]. Background adjustment, quantile normalization and probe set summarization were performed using the GC-RMA algorithm (Bioconductor gcrma for R package, Version 2.3). MIAME compliant data sets are deposited in the ArrayExpress database (accession number: E-MEXP-3917; http://www.ebi.ac.uk/arrayexpress). The snap frozen specimens for the microarray investigations were partly used from different dogs, but with an intersection of 4 dogs which were investigated in both the microarray and immunofluorescence study. For the microarray investigations, tissue of dogs with only one type of lesion (acute, subacute, chronic) within the cerebellum was used.

For microarray analysis of TMEV-DL, RNA was isolated from snap frozen spinal cord specimens using the RNeasy Mini Kit (Qiagen, Hilden, Germany), amplified and labelled employing the MessageAmp II Biotin Enhanced Kit (Ambion, Austin, TX, USA) and hybridized to GeneChip mouse genome 430 2.0 arrays (Affymetrix, Santa Clara, CA, USA) as previously described [37]. Background adjustment and quantile normalization was performed using RMAexpress [71]. MIAME compliant data sets are deposited in the ArrayExpress database (E-MEXP-1717; http://www.ebi.ac.uk/arrayexpress). Snap frozen spinal cord specimens for the microarray investigations were used from the same animals that were investigated by immunofluorescence. However, tissue from time point 245 dpi was included in the immunofluorescence study only, while microarray data derived from 14, 42, 98, and 196 dpi.

A comparable bottom-up analysis of manually selected candidate genes was done for CDV-DL and TMEV-DL using classical statistics as previously described [72]. Accordingly, 87 canine and 93 mouse candidate genes subclassified based on their association with (1) Mitochondrial respiratory chain, (2) NADPH oxidase system, (3) ROS detoxification system, (4) pyruvate dehydrogenase complex, (5) ROS toxification system, and (6) catabolic enzymes with oxidase activity were manually extracted from the canonical oxidative phosphorylation and leukocyte transendothelial migration pathways of the Kyoto Encyclopedia of Genes and Genomes (KEGG) database (Entry: map00190 and hsa04670) and peer-reviewed published literature [73,74,75,76,77,78,79,80]. The normalized expression values of these genes were evaluated for significant differences between the groups employing a Kruskal–Wallis test followed by independent pairwise Mann–Whitney U-tests for CDV and independent pairwise Mann–Whitney U-tests for TMEV, respectively (IBM SPSS Statistics, Version 20). Statistical significance was designated as *p* ≤ 0.05.

Intersections of the differentially expressed genes of CDV-DL and TME were compared between the two models using Venn diagrams (Oliveros, J.C. (2007–2015) Venny). An interactive tool for comparing lists with Venn’s diagrams (http://bioinfogp.cnb.csic.es/tools/venny/index.html) in order to reveal commonalities and differences between the gene expression in both animal models.

## 5. Conclusions

In summary, the presented study demonstrated markedly increased amounts of ROS-induced metabolites within intralesional oligodendrocytes, microglia, and macrophages in all subgroups of CDV-DL, highlighting that ROS-induced membrane damage represents an early key event in the pathogenesis of demyelination in this disease. Furthermore, the transcriptome analysis suggests that the NADPH oxidase system could potentially function as the main producer of ROS in both CDV-DL and TMEV-DL. This might point to the possibility that in agreement with the bystander demyelination hypothesis most of the ROS-production in CDV-DL is attributed to activated microglia/macrophages. Further focusing on microglia/macrophages and ROS generation in the pathogenesis of CDV-DL appears as a promising target for future studies. In contrast to CDV-DL, TMEV infection in mice showed an increase of 8OHdG and only low amounts of MDA. These results substantially differ from MS [11,40]. In contrast to the ROS products antioxidant enzymes were only present in low amounts in astrocytes but were clearly present in macrophages and microglia and oligodendrocytes. Though there were robust differences between the amounts of ROS-metabolites in CDV-DL and TMEV-DL, the transcriptome data showed surprising similarities with genes of the NADPH oxidase enzyme system being similarly upregulated in TMEV-DL (up to 28.6%) and CDV-DL.

The present findings do not allow a final conclusion on the causative role of ROS during the demyelinating disease process but provide a substantial basis future research on the role of ROS in both models. CDV-DL showed a substantially higher amount of ROS products in contrast to TMEV-DL. This may indicate that focusing on the role of ROS—CDV-DL more closely mimics ROS induced damage in MS and is the more appropriate model for future studies on the pathogenetic role of ROS during demyelinating disease. However, both animal models also differ in the presence of ROS and antioxidants products within astrocytes as compared to reported findings in MS [11,45,81].

## Figures and Tables

**Table 1:** Differential expression of manually selected genes functionally associated with the generation and detoxification of ROS in CDV-DL.

Function	Genes (N)	Differentially Expressed Genes			
		Direction of change	2 vs. 1	3 vs. 1	4 vs. 1
Mitochondrial respiratory chain	50	Up	0	0	0
		Down	7 (14%)	17 (34%)	6 (12%)
NADPH oxidase enzyme system	13	Up	5 (38.5%)	4 (30.8%)	2 (15.38%)
		Down	0	0	0
ROS detoxification system	12	Up	3 (25%)	3 (25%)	1 (8.33%)
		Down	1 (8.33%)	1 (8.33%)	0
Catabolic enzymes with oxidase activity	3	Up	1 (33.33%)	1 (33.33%)	1 (33.33%)
		Down	0	1 (33.33%)	1 (33.33%)
Pyruvate dehydrogenase complex)	5	Up	0	0	0
		Down	0	1 (20%)	0
ROS toxification system	4	Up	0	0	0
		Down	0	0	0

**Table 2:** Differential expression of manually selected genes functionally associated with the generation and detoxification of ROS in TMEV-DL.

Function	Genes (N)	Differentially Expressed Genes				
		Direction of change	14dpi	42dpi	98dpi	196dpi
Mitochondrial respiratory chain	55	Up	0	0	0	0
		Down	7(12.7%)	31(56.36%)	30(54.55%)	36 (65.45%)
NADPH oxidase enzyme system	9	Up	5 (55.5%)	6 (66.7%)	6 (66.7%)	6 (66.7%)
		Down	0	0	0	0
ROS detoxification system	17	Up	0	2 (11.76%)	1 (5.88%)	4 (23.53%)
		Down	1 (5.88%)	2 (11.76%)	3 (17.65%)	3 (17.65%)
Catabolic enzymes with oxidase activity	3	Up	1 (33.3%)	2 (66.7%)	2 (66.7%)	1 (33.3%)
		Down	0	1 (33.3%)	0	0
Pyruvate dehydrogenase complex)	5	Up	0	0	0	0
		Down	0	3 (60%)	2 (40%)	0
ROS toxification system	4	Up	0	0	0	0
		Down	0	0	1 (25.0%)	1 (25.0%)

## Supplementary Materials

Supplementary materials can be found at https://www.mdpi.com/1422-0067/20/13/3217/s1.

## Abbreviations

CDV	Canine distemper virus
CDV-DL	Canine distemper virus -induced demyelinating leukoencephalitis
TME	Theiler’s murine encephalomyelitis
TMEV-DL	Theiler’s murine encephalomyelitis virus—induced demyelinating leukomyelitis
TMEV	Theiler’s murine encephalomyelitis virus
MS	Multiple Sclerosis
ROS	Reactive oxygen species
NADPH	Nicotinamide adenine dinucleotide phosphate
ATP	Adenosine triphosphate
MDA	Malondialdehyde
8-OHdG	8-hydroxydeoxyguanosine
8-OHG	8-hydroxyguanosine
CNS	Central nervous system
PUFAs	Polyunsaturated fatty acids
SOD	Superoxide dismutases
MnSOD, aka SOD2	Manganese SOD
O_2_^• −^	superoxide
H_2_O_2_	hydrogen peroxide
CSF	Cerebrospinal fluid
LFB	Luxol fast blue
dpi	Days post infection
CNPase	2′,3′-cyclic-nucleotide 3′-phosphodiesterase
GFAP	Glial fibrillary acidic protein
Iba-1	Ionized calcium-binding adapter molecule 1
ND5	NADH dehydrogenase subunit 5
RAC2	Ras-related C3 botulinum toxin substrate 2
CYBB	Cytochrome b-245, beta polypeptide
XDH	Xanthine dehydrogenase
NOX2	NADPH oxidase
EAE	experimental autoimmune encephalomyelitis
HE	Hematoxylin and eosin
LFB	Luxol fast blue
PBS	Phosphate buffered saline
FCE	Fibrocartilaginous embolus
KEGG	Kyoto Encyclopedia of Genes and Genomes
